# Fish protein intake is a novel dietary approach for managing diabetes‐associated complications in diabetic Wistar rat model

**DOI:** 10.1002/fsn3.2069

**Published:** 2020-12-23

**Authors:** Humaira Muzaffar, Muhammad Naeem Faisal, Haseeb Anwar, Abid Hussain, Junaid Ali Khan, Faqir Muhammad, Bilal Aslam, Aisha Mahmood, Ahmed Abdelsadik, Jawad Aslam, Muhammad Faisal Manzoor, Nazir Ahmad, Emad Karrar

**Affiliations:** ^1^ Institute of Pharmacy, Physiology and Pharmacology University of Agriculture Faisalabad Pakistan; ^2^ Department of Physiology Government College University Faisalabad Pakistan; ^3^ School of Food Science and Engineering South China University of Technology Guangzhou China; ^4^ Department of Physiology and Biochemistry Cholistan University of Veterinary and Animal Sciences Bahawalpur Bahawalpur Pakistan; ^5^ Department of Zoology Aswan University Aswan Egypt; ^6^ School of Food and Biological Engineering Jiangsu University Zhenjiang China; ^7^ Institute of Home and Food Sciences Government College University Faisalabad Pakistan; ^8^ Department of Food Engineering Faculty of Engineering University of Gezira Wad Medani Sudan

**Keywords:** alloxan‐induced diabetic rat model, diabetes‐associated complications, fish protein, novel dietary approach

## Abstract

Diabetes mellitus is a metabolic disorder associated with short term as well as long‐term undesirable complications caused by persistent hyperglycemia. Recently, there has been emerging evidence that natural foods and their bioactive compounds are the key contributors to the treatment of diabetes and associated complications. This study was designed to explore the therapeutic efficacy of a fish protein‐rich diet for managing diabetes and associated complications in the diabetic Wistar rat model. A high‐protein (HP) diet (45% and 55% fish protein rich in ω3 fatty acids) was given to alloxan‐induced diabetic rats for 28 days. Blood samples were collected for monitoring serum glucose, oxidative stress markers, lipid profile, kidney function markers, serum proteins, and liver function markers. Results indicated that there was a noteworthy control (*p* < .05) of serum glucose, oxidative stress, and lipid profile in HP diet treated diabetic rats. Treatment with 45% and 55% fish diet appreciably improved the concentration of serum creatinine, urea, uric acid and exhibited a vibrant improvement in renal functions. Our results confirmed that the HP diet restored total protein and albumin concentration in blood. The HP diet treatment also restored the normal serum aspartate transaminase and alanine aminotransferase concentration.

## Graphical abstract

Diet acquiring the key role in metabolic homeostasis and promoting health. A balanced diet based on type, amount, and macronutrients such as proteins, fats, and carbohydrates is vital for managing diabetes. Results revealed the protective and therapeutic efficacy of high‐protein diet (fish protein) in alloxan‐induced diabetic rat models through normalizing serum glucose concentration, oxidative stress, lipid profile, RFTs, and LFTs. Conclusively, our findings suggest that fish protein can be useful in the management of diabetes and associated complications by medical nutrition. However, further research studies are required to further elucidate its mode of action.

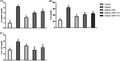

## 1 INTRODUCTION

Diabetes is a metabolic syndrome associated with hyperglycemia, β‐cell dysfunction, impaired secretion of insulin, or developing insulin resistance (Mirmiran et al., 2014; Muzaffar et al., 2019; Rehman et al., 2018). Numerous metabolic ailments including subclinical inflammation, oxidative stress, impaired lipoprotein, and lipid metabolism, hypertension, and vascular endothelial dysfunction are usually accompanied by diabetes (Bekyarova et al., 2007; Mooradian, 2009). Leading to long‐term pathogenic complications such as macro‐ and microvascular complications including nephropathy, retinopathy, and neuropathy, resulting in increased mortality rate as well as decreased quality of life (Constantino et al., 2013). Although many pharmacological interventions are available for managing diabetes such as insulin therapy and oral hypoglycemic agent, an alarmingly increasing trend in incidents of undesirable complications among diabetic patients is currently evident (Mirmiran et al., 2014; Santaguida et al., 2005).

In medical science, nutritional therapy is considered one of the key players for the management of diabetes. The main components of the diet for diabetic patients are an estimation of nutrients according to energy requirements, counting of carbohydrates, total glycemic load, and index. Common important recommendations for a healthy diet including dietary intake of protein, cholesterol, and fats (Dämon et al., 2011; Naseem et al., 2020; Yasin et al., 2020); however, it is not yet clear whether this dietary approach is adequate for preventing long‐term diabetes‐associated complications (Mirmiran et al., 2014). Administration of different supplements such as antioxidants, vitamins, ω3 fatty acids, fibers, herbs, and other nutraceutical has also been recommended for the control of hyperglycemia but insufficient data are available in the literature to support these recommendations (Bahadoran et al., 2012; Perera & Li, 2012). The therapeutic properties of foods may be a missing step during the process of medical nutritional therapy and it could boost the efficiency of dietary management of diabetic patients. Therefore, the present study was designed to evaluate the potential of a high‐protein diet (fish protein rich in ω3 fatty acids) for managing diabetes and associated complications.

## 2 MATERIALS AND METHODS

### 2.1 Subjects and induction of diabetes

Wister albino rats weighing about 180–250 g were used for this research work. The rats were kept at the animal house facility, University of Agriculture Faisalabad. The research trial was carried out by strictly following the guidelines on the use and care of animals, approved by the institutional Bioethics Review Committee University of Agriculture, Faisalabad, Pakistan (which regulates the implementation of Biosafety/Bioethics protocols at University of Agriculture, Faisalabad under the guidelines of National Biosafety Committee and Punjab Biosafety Rules 2014; Ref.#UAF/ORIC/7141). An acclimatization period of about two weeks was provided to the rats before the start of the experiment under standard conditions, that is, Temp = 26 ± 2°C and humidity = 40%–60%. During the acclimatization period, all the rats received a routine diet and water ad libitum (Table 1). The rats were divided randomly into the following five groups as described in Table 2.

**TABLE 1:** Diet composition (g/kg) and nutrient levels on dry matter (DM) basis (%)

Ingredients	Amount(g/kg)
Corn starch	230
Maltodextrine	100
Sucrose	100
Soya bean meal	420
Maize bran	50
Soybean oil	50
Methionine	3
AIN 93G Vit Mix	10
AIN 93G Min Mix	35
Choline bitartrate	2
Total calories (kcal)	3,822

**TABLE 2:** Study design for dietary intervention

Groups	Diet	Group name
Group I	Standard diet	Control
Group II	Alloxan‐induced diabetic rats fed on a standard diet	Diabetic
Group III	Alloxan‐induced diabetic rats treated with hypoglycemic drug glibenclamide fed on a standard diet	Diabetic + glib.
Group IV	Alloxan‐induced diabetic rats receiving 45% high‐protein diet (HPD)	Diabetic + HPD 45%
Group V	Alloxan‐induced diabetic rats receiving 55% high‐protein diet	Diabetic + HPD 55%

Before the induction of diabetes, the blood glucose levels of rats were determined. Diabetes was induced by administering a single injection (intraperitoneal) of freshly prepared alloxan monohydrate (dissolved in normal saline) at 140 mg/kg body weight (Federiuk et al., 2004). The fasting blood glucose level of each rat was measured from the tail vein by using a glucometer available commercially (OnCall*^®^ Ez II*; SN 303S0014E09) after 3, 7, and 10 days of alloxan injection to determine whether the animals had become diabetic or not. The rats exhibiting a fasting blood glucose level of ≥250 mg/dl were included in this research project. After ten days of alloxan administration, a high‐protein (HP) diet, that is, 45% and 55% fish was provided to group IV and V, respectively, for a period of the next 28 days.

### 2.2 Blood sampling

All the rats were decapitated by cervical dislocation at the end of an experimental trial. Blood samples were collected and centrifuged at 3,000 *g* for 10–15 min to separate serum. Then, the separated serum was stored at −20°C to be used for further biochemical analysis.

### 2.3 Serum glucose

A commercially available kit (Bioclin^®^ Glucose Monoreagent diagnostic kit) was used for the determination of serum glucose level by following the instructions as indicated in instruction manual of assay kit.

### 2.4 Oxidative stress markers

The calorimetric method as explained by Erel (2005), after slight modifications, was used to determine serum total oxidative status (TOS) and the total antioxidant capacity (TAC) in the serum samples.

### 2.5 Serum lipid profile

The total cholesterol and triglycerides concentration was determined by using the Dia‐Sys Diagnostic Systems reagent kit method. The commercially available reagent kit (Randox Laboratories Ltd.) method was used to determine HDL‐Cholesterol level in serum. The concentration of LDL cholesterol in serum was calculated with the help of the following formula:

$$\text{LDL - cholesterol}\;\left(\text{mg/dl}\right)=\text{Total cholesterol}‐\left[\text{Triglycerides/}5+\text{HDL - Cholesterol}\right]$$

### 2.6 Renal function test

The concentration of creatinine, urea, and uric acid in serum was determined spectrophotometrically by the use of commercially available kits (Bioclinc^®^ Creatinine Kinetic diagnostic Kit; K067; Bioclinc^®^ Urea UV Kinetic diagnostic Kit; K056; Bioclinc^®^ Uric Acid Monoreagent diagnostic Kit; K139) following the instructions as indicated by manufacturers in instruction manuals.

### 2.7 Serum proteins

Commercially available kits (Bioclin^®^ Total Protein Monoreagent Diagnostic Kit; K031 and Bioclin^®^ Albumin Monoreagent Diagnostic Kit; K040) were used to determine total protein and albumin concentration in serum samples by following the manufacturer's instructions. Serum globulin concentration in each sample was estimated by subtracting the value of serum albumin concentration from total protein concentration.

### 2.8 Liver function markers

Commercially available kits (Bioclin^®^ Transaminase AST kinetic diagnostic kit; K048 and Bioclin^®^ Transaminase ALT kinetic diagnostic kit; K049) were used for determining the serum AST (Aspartate transaminase) and serum ALT (Alanine transaminase) levels according to the method described by IFCC (1980) using an automated biochemical analyzer with assay kits.

### 2.9 Statistical analysis

Obtained results were subjected to statistical analysis by applying analysis of variance (ANOVA), which means were then compared by DMR (Steel, 1997) test, and data were expressed as a mean ± *SE*. Statistical tests were performed by using Graph Pad prism 6 and CoStat computer software.

## 3 RESULTS AND DISCUSSION

Table 3 illustrates the effect of fish protein diet on serum glucose concentration, serum total oxidative status (TOS), and serum TAC in alloxan‐induced diabetic rats. Alloxan monohydrate significantly (*p* < .05) increased the mean serum glucose concentration in the positive control (diabetic) group as compared to negative control and all other treatment groups. Groups treated with glibenclamide and high‐protein diet exhibited a significant reduction (*p* < .05) in the concentration of serum glucose. The difference between groups fed 45% and 55% protein diet was statistically nonsignificant when compared with each other. This finding indicated congruence with a previous study in which a decline in fasting blood glucose level was observed after the consumption of cod protein in normoglycemic rats (Lavigne et al., 2000).

**TABLE 3:** Effect of fish protein diet on serum glucose concentration, serum total oxidative status (TOS), and serum total antioxidant capacity (TAC) in alloxan‐induced diabetic rats

Groups	Serum Glucose (mg/dl ± *SE*)	Serum TOS (μmol/L ± *SE*)	Serum TAC (mmol/L ± *SE*)
Control	91.26 ± 5.19^C^	12.74 ± 0.84^B^	2.41 ± 0.11^A^
Diabetic	606.8 ± 12.3^A^	24.40 ± 0.67^A^	1.08 ± 0.21^B^
Diabetic + glib.	178.1 ± 8.79^B^	13.99 ± 1.24^B^	2.74 ± 0.34^A^
Diabetic + HPD 45%	163.8 ± 22.2^B^	14.37 ± 1.78^B^	3.07 ± 0.18^A^
Diabetic + HPD 55%	198.1 ± 11.5^B^	13.09 ± 1.06^B^	3.22 ± 0.51^A^

Note

Data are presented as mean ± *SE* at *p* < .05 using one‐way ANOVA followed by DMR.

Results revealed that the mean serum concentration of TOS (Table 3) was significantly higher (*p* < .05) in the positive control diabetic group as compared to negative control and all treatment groups. Glibenclamide and both high‐protein diet treated groups exhibited a significant reduction (*p* < .05) in serum TOS level. However, high‐protein diet groups fed 45% and 55% of fish protein showed the nonsignificant differences when compared with each other.

The level of TAC (Table 3) exhibited a significant difference (*p* < .05) among negative control, positive control (diabetic), and all treatment groups. Diabetes induction by alloxan monohydrate significantly (*p* < .05) reduced the mean serum TAC concentration in positive control as compared to negative control and all treatment groups. Glibenclamide and high‐protein diet treatment for 28 days showed a significant rise (*p* < .05) and restored serum TAC level.

Results of the current study exhibited that induction of diabetes by alloxan triggered a noticeable increase (*p* < .05) in TOS leading to pancreatic β‐cell damage in the pancreas of the positive control group than that of the negative control group. Apoptotic cell death of the β‐cells of the pancreas due to alloxan‐induced overproduction of ROS (reactive oxygen species) has also been observed in previous research studies (Almalki et al., 2016; Mokhtari & Mohammadi, 2012). Superoxide anion induced oxidative stress is supposed to be involved in the pathogenesis of alloxan‐induced diabetes (Kaneto et al., 2007). However, intake of HP diet in hyperglycemic rats for 28 days potentially enhanced the TAC and significantly reduced (*p* < .05) the oxidative stress. These effects are due to the antioxidant potential and free radical scavenging activity of the HP diet. So, the intake of the HP diet in diabetic rats fortified their antioxidant system leading to improved capability to manage oxidative stress and thus, helping to cope up with the subsequent damage of β‐cells. These results of the current study endorsed by the findings of previously conducted research studies where the intake of antioxidants reduced hyperglycemia and diabetic complications triggered by oxidative stress (Thomson et al., 2007).

**FIGURE 1:**
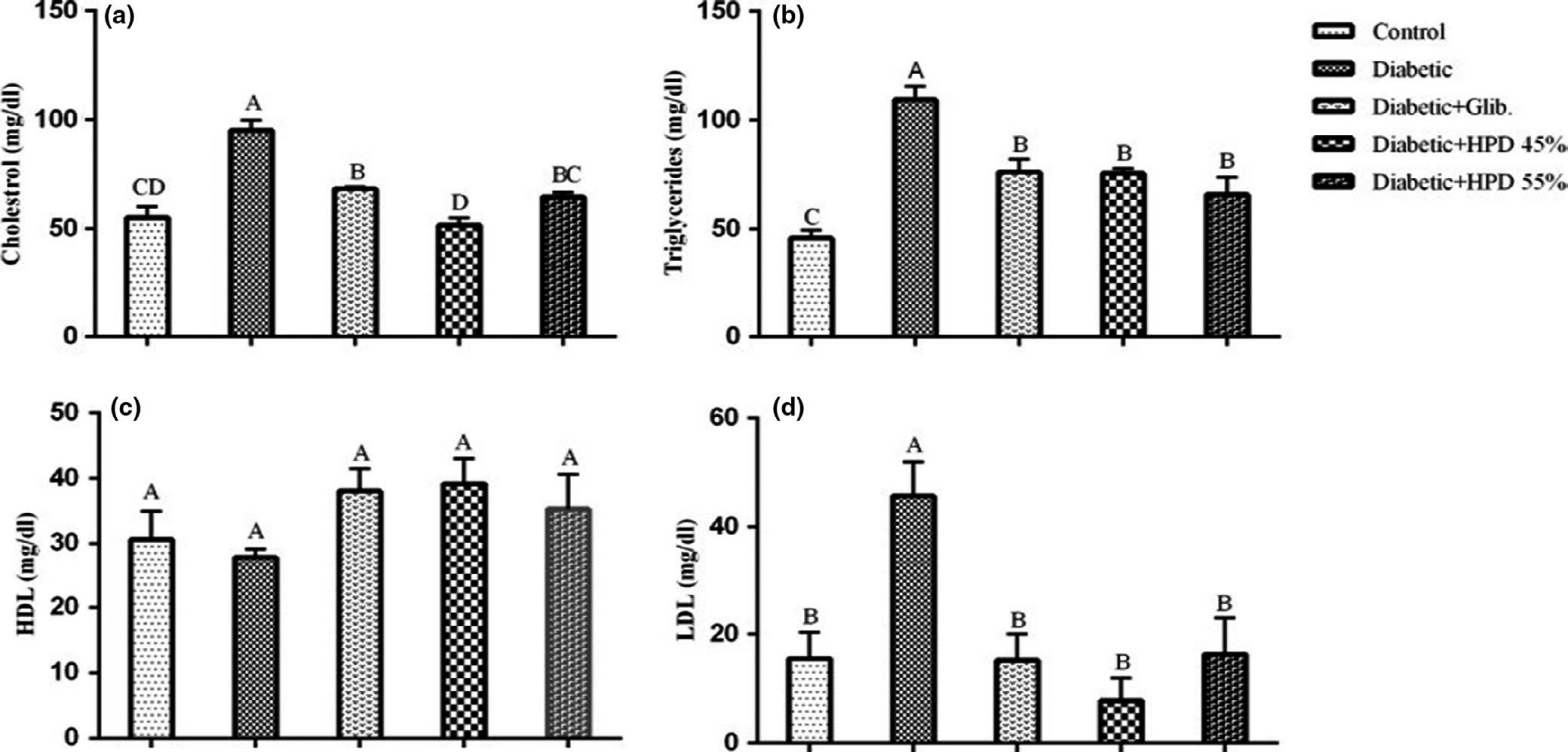
Effect of high‐protein diet on lipid profile (a) total cholesterol (mg/dl) (b) triglycerides (mg/dl) (c) HDL (mg/dl) (d) LDL (mg/dl) in alloxan‐induced diabetic rats. Data are presented as mean ± *SE* at *p* < .05 using one‐way ANOVA followed by DMR

Lipid profile results showed that the mean values of serum total cholesterol and triglycerides were significantly (*p* < .05) higher in diabetic rats as compared to negative control and all other treatment groups (Figure 1). Groups treated with glibenclamide and with a high‐protein diet exhibited a significant (*p* < .05) reduction and restored normal serum cholesterol and triglycerides levels (Figure 1). Statistical analysis of serum HDL revealed a nonsignificant effect of different groups. The mean values of serum HDL (mg/dl ± *SE*) were slightly increased in glibenclamide and high‐protein diet treated groups as compared to diabetic and negative control groups but this difference was statistically nonsignificant (*p* < .05). The mean value of serum LDL (mg/dl) was significantly (*p* < .05) higher in diabetic rats than that of the negative control group. Glibenclamide and both high‐protein diet treatment for 28 days led to a significant reduction in serum LDL levels after diabetes induction. However, the difference between groups fed 45% and 55% protein diet was nonsignificant when compared with each other.

One of the most frequent and common complications of diabetes mellitus is hyperlipidemia. In the current study, a noticeable rise (*p* < .05) in triglycerides, LDL cholesterol, and total cholesterol while a reduction in HDL cholesterol was observed in rats from the positive control group. It was also observed in previous research studies that reduced insulin level, fails to activate the lipoprotein lipase enzyme, thus leading to hyperlipidemia (Puntel et al., 2005; Verma et al., 2013). There was a remarkable control (*p* < .05) of lipid profile was seen in the HP diet treated diabetic rats. It is observed that the lipid‐lowering capability of the HP diet is associated with ameliorate hyperglycemia. The provision of essential amino acids present in the HP diet (fish protein) might help in the amelioration of oxidative stress of diabetes. Moreover, dietary fibers present in the HP diet could also be one of the main factors responsible for lowering blood cholesterol levels by increasing its excretion as well as decreasing its absorption.

**FIGURE 2:**
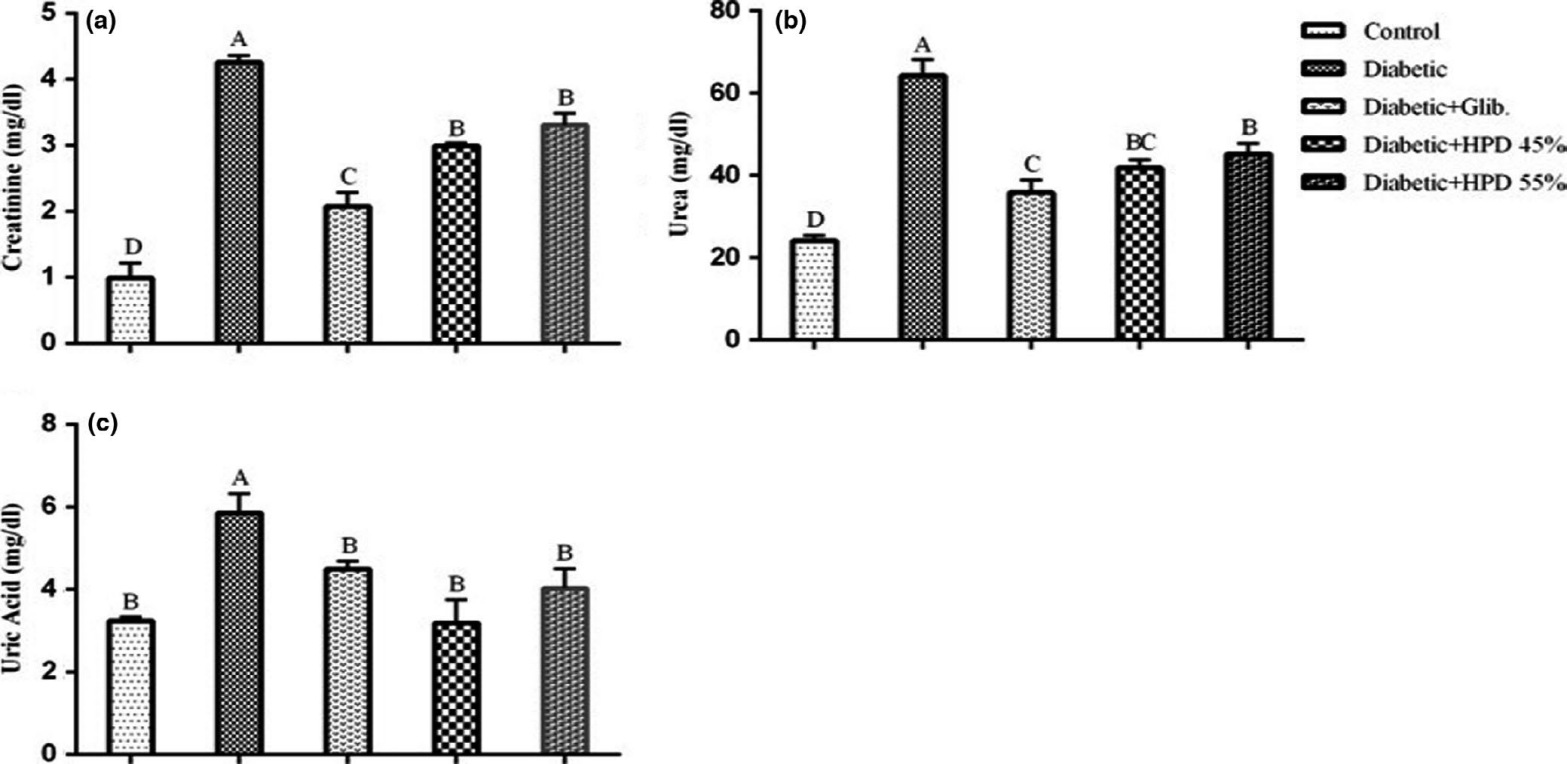
Effect of high‐protein diet on renal function markers (a) serum creatinine (mg/dl ± *SE*) (b) serum urea (mg/dl ± *SE*) (c) serum uric acid (mg/dl) in alloxan‐induced diabetic rats. Data are presented as mean ± *SE* at *p* < .05 using one‐way ANOVA followed by DMR

The effect of a high‐protein diet on renal function markers is presented in Figure 2. Alloxan‐induced diabetic group exhibited considerably (*p* < .05) higher concentrations of serum creatinine, urea, and uric acid as compared to negative control and all other treatment groups. Glibenclamide and high‐protein diet treated groups exhibited a substantial (*p* < .05) decline in serum levels of urea, uric acid, and creatinine that showed improvement in renal function by protein diet. The overall mean for renal function markers did not differ significantly between 45% and 55% protein diet‐treated groups.

The persistent hyperglycemic condition leads to an increase in serum urea, uric acid, and creatinine levels which are ultimately responsible for the impairment of renal functions. Results revealed significant increased (*p* < .05) concentration of serum urea, uric acid, and creatinine in untreated diabetic rats while treatment with HP diet (fish protein) reduced the levels of renal function markers that might help to protect the renal system from injury (Naseem et al., 2016) of diabetes by alloxan monohydrate account for a noticeable reduction in serum protein contents due to stimulation of gluconeogenesis. Increased breakdown of both plasma and hepatic proteins might be responsible for increased levels of uric acid and urea in the serum of untreated diabetic rats (Hassan et al., 2009). Treatment with 45% and 55% fish diet appreciably improve the levels of serum urea and total protein as well as showed a vibrant improvement in renal functions probably due to its antioxidant potential (Gandhi & Sasikumar, 2012).

**FIGURE 3:**
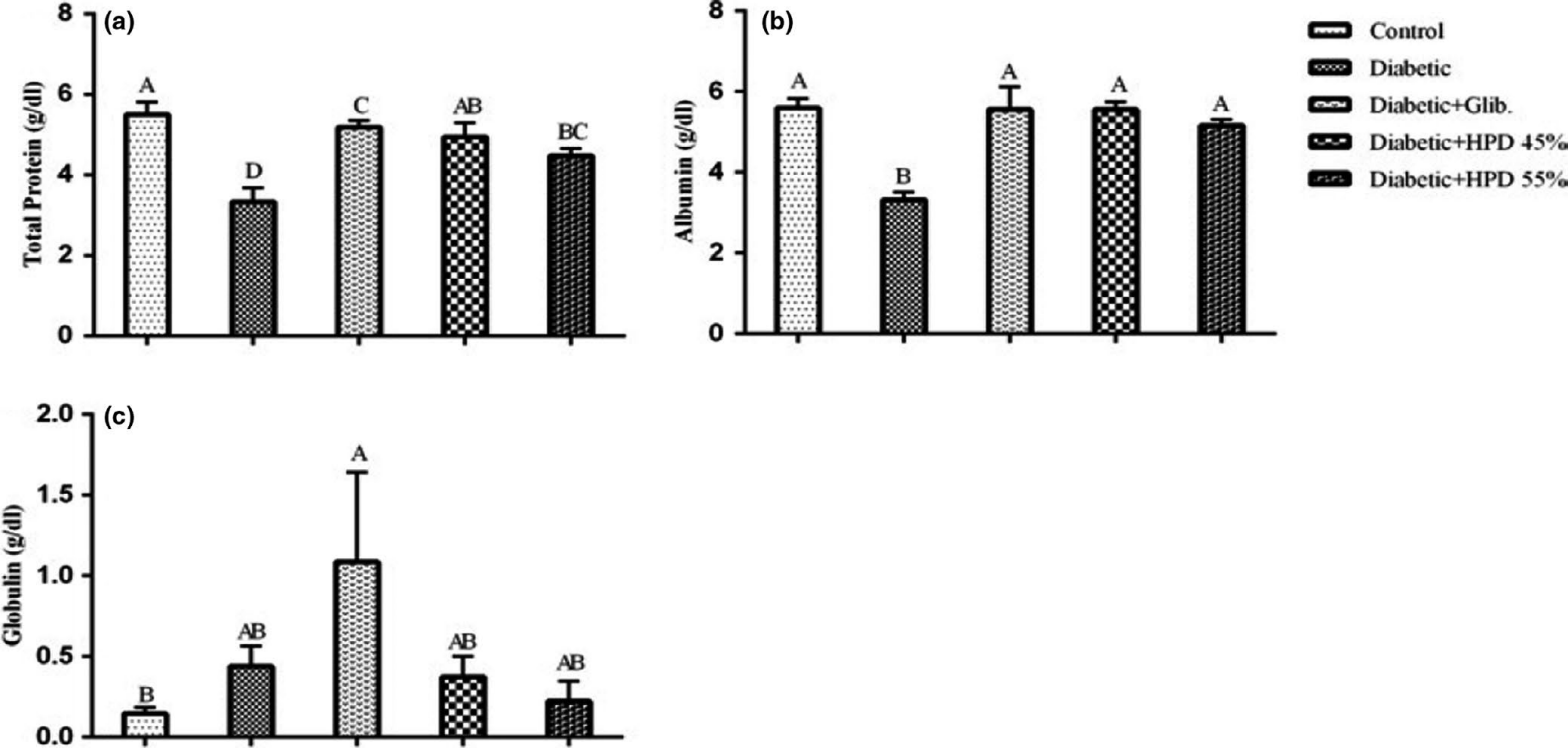
Effect of high‐protein diet on serum proteins (a) serum total protein (g/dl) (b) serum albumin (g/dl) (c) serum globulin (g/dl) in alloxan‐induced diabetic rats. Data are presented as mean ± *SE* at *p* < .05 using one‐way ANOVA followed by DMR

Figure 3 shows the effect of a high‐protein diet on serum proteins in alloxan‐induced diabetic rats. Results revealed after statistical analysis that a significant difference in total protein levels of negative control, positive control, and all treatment groups. The total protein and albumin concentration (mean ± *SE*) was appreciably (*p* < .05) reduced in the diabetic group in comparison with the negative control group. Glibenclamide and high protein diet treated groups presented a noteworthy (*p* < .05) rise and restored normal serum total protein and albumin levels. However, a nonsignificant difference between groups fed 45% and 55% protein diet was observed when compared with each other. Results for serum globulin revealed a nonsignificant difference in globulin levels of negative control, diabetic, glibenclamide, and high protein diet treated groups. The serum globulin concentration was higher in glibenclamide‐treated groups as compared to the negative control group while all other groups showed a slight increase in globulin level and this increase was statistically nonsignificant (*p* < .05).

The main clinical indicators of diabetic nephropathy include microproteinuria and albuminuria due to increased protein catabolism associated with diabetes mellitus (Elmarakby & Sullivan, 2012). Results revealed that alloxan monohydrate pointedly decreased (*p* < .05) the concentration of serum albumin, globulin, and total proteins in untreated diabetic rats as compared to negative control and treated groups. The level of serum proteins depends on the balance between protein synthesis and its catabolism in the body. Reduction in serum albumin and total protein levels might be due to enhanced catabolism of protein associated with diabetes (Alagammal et al., 2012) leading to albuminuria and microproteinuria that are considered to be the main clinical indicators of diabetic nephropathy (Elmarakby & Sullivan, 2012). Our results confirmed that diabetic rats receiving HP diet restored total protein and albumin levels in serum possibly because of the insulin‐mediated tissue uptake of amino acids, increase in protein synthesis, and decreased degradation of tissue protein (Vidhya & Udayakumar, 2016).

**FIGURE 4:**
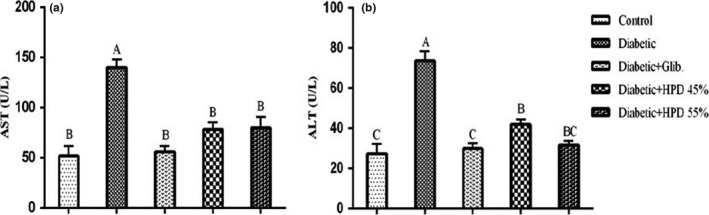
Effect of high‐protein diet on liver function markers (a) serum aspartate transaminase (AST; U/L) (b) serum alanine transaminase (ALT; U/L) in alloxan‐induced diabetic rats. Data are presented as mean ± *SE* at *p* < .05 using one‐way ANOVA followed by DMR

Figure 4 represents the effect of a high‐protein diet on liver function markers The mean AST and ALT concentration was considerably raised (*p* < .05) in diabetic rats as compared to the negative control group. Glibenclamide and high‐protein diet treatment for 28 days resulted in a significant reduction (*p* ≤ .05) and restoration in normal serum AST and ALT levels.

The liver is the key player in carbohydrate metabolism and its storage in the form of glycogen and also involved in gluconeogenesis by utilizing noncarbohydrate sources. Metabolic disorders especially diabetes mellitus can cause damage to hepatocytes (Ni et al., 2012), hence, the release of intracellular elements including liver enzymes into the systemic circulation (Mahmood et al., 2019). Measurement of liver enzymes in the serum may serve as a valuable means for the diagnosis of liver damage. Results revealed a significant rise (*p* ≤ .05) in the serum aspartate transaminase (AST) and alanine transaminase (ALT) levels in alloxan‐induced diabetic rats as compared to the negative control group possibly due to the out‐flow of hepatic enzymes from damaged hepatocytes into plasma. The treatment with the HP diet restored the normal serum AST and ALT concentration (Collins et al., 2016).

## 4 CONCLUSION

Diet acquiring a key role in metabolic homeostasis and promoting health. A balanced diet based on type, amount, and macronutrients such as proteins, fats, and carbohydrates is vital for managing diabetes. Results revealed the protective and therapeutic efficacy of a high‐protein diet (fish protein) in alloxan‐induced diabetic rat models through normalizing serum glucose concentration, oxidative stress, lipid profile, RFTs, and LFTs. Conclusively, our findings suggest that fish protein can be useful in the management of diabetes and associated complications by medical nutrition. However, further research studies are required to further elucidate its mode of action.

## CONFLICT OF INTEREST

The authors declare that they have no conflict of interest.

## Data Availability

The dataset supporting the conclusions of this article is included within the article.
